# Influence of Contralateral Hip Status on Pelvic Tilt After Total Hip Arthroplasty

**DOI:** 10.1016/j.artd.2024.101460

**Published:** 2024-08-05

**Authors:** William Oetojo, Patrick Lawler, James Padley, Jim Pierrepont, Daniel Schmitt, Nicholas Brown

**Affiliations:** a Stritch School of Medicine, Loyola University, Maywood, IL; b Corin Group, Cirencester, UK; c Department of Orthopaedic Surgery and Rehabilitation, Loyola University Health System, Maywood, IL

**Keywords:** Pelvic tilt, Acetabular cup, Contralateral hip, Postoperative, Standing x-ray, Hip arthroplasty

## Abstract

**Background:**

Every degree of change in pelvic tilt (PT) leads to a 0.7° change in anteversion and a 0.3° change in inclination. This study aimed to determine the significance of contralateral hip arthritis on changes in PT using preoperative and postoperative anteroposterior radiographs.

**Methods:**

There were 193 primary total hip arthroplasties done by 2 surgeons at a single academic tertiary referral center reviewed between September 2021 and January 2023. PT was calculated as Tilt = −(ln[(B/A) × (1/0.483)]) / 0.051. Value A is the distance from the base of the SI joint to the superior margin of the obturator foramen; value B is the height of the obturator foramen. After exclusions, contralateral hips were identified as being normal (n = 75), arthritic (n = 39) (Tönnis grade 3/4), replaced (n = 34), or having undergone simultaneous bilateral total hip arthroplasty (n = 5) on postoperative films. Difference in PT was measured between preoperative and postoperative films taken 1-3 months after surgery. Analyses for statistical significance were calculated using *t*-tests and one-way analysis of variance.

**Results:**

Average change in PT in patients with normal contralateral hips was −5.2° with an absolute mean difference of 7.6°, −1.5° for arthritic contralateral hips with an absolute mean difference of 5.0°, −1.6° for replaced contralateral hips with a mean absolute difference of 4.3°, and 2.2° for bilateral hips with a mean absolute difference of 2.6° (*P* < .01).

**Conclusions:**

Differences in postoperative PT changes between healthy, arthritic, and replaced contralateral hip study groups were significant. Changes in preoperative to postoperative tilt may have implications for optimal cup placement.

## Introduction

Pelvic tilt (PT) is known to have significant effects on gait, posture, and functional acetabular cup position [[1], [2], [3], [4], [5], [6], [7]]. Total hip arthroplasty (THA) has been shown to significantly alter a patient’s PT postoperatively. Specifically, patients have been noted to develop a dramatically increased posterior tilt after THA [[8], [9], [10], [11]]. Ishida et al noted that patients with severe preoperative anterior PT exhibited posterior changes in PT after THA and had poorer functional outcomes after 1 year compared to patients with preoperative posterior PT [9]. Follow-up at 2-4 years have also shown significant posterior tilting after THA [12]. Katsura et al followed the progression of posterior tilting and found that the first year after THA held the most significant change; progression was also more rapid in patients with femoral neck fractures and subchondral fractures of the femoral head [9]. Despite most studies documenting consistent posterior tilting, Blondel et al noted no significant variation in PT between preoperative tilt and PT after 3-year follow-up (n = 50) [13].

One possible theory to the reasons for posterior tilting after THA may include the release of hip flexor muscles or contracted anterior capsule. Increased activity of hip flexor muscles relative to hip extensors is associated with increased anterior PT [14]. Thus, without compensatory hip flexion against hip extensors, posterior tilting could develop after THA. Rossi et al also identified an accelerated rate of strength recovery in hip extensor muscles after THA relative to hip flexor strength [15].

Of note, osteoarthritis (OA) has been identified as one of many culprits in PT variations [1,11,16,17]. Significant postoperative changes in PT after THA have been seen in patients with primary and secondary hip OA [11,16], with postoperative PT changes likely being influenced by preoperative PT [16]. Huang et al also found that compensation in gait patterns from medial knee OA has led to increased anterior tilting, supporting the notion that OA plays a part in the multifactorial changes in PT [17].

To our knowledge, there has only been one other study that specifically looked into changes in postoperative PT changes with consideration of contralateral hip conditions [11]. Nishiwaki et al found that greater posterior PT was noted in patients with normal contralateral hips when compared to joint space narrowing/artificial joints in the contralateral side (<2% tilted more than 5 degrees for OA and were replaced vs 15% tilted more than 5 degrees in normal contralateral hip). Their theory was that the lack of contracture in the opposite hip joint in conjunction with surgical release of the operative hip led to a greater influence on PT change in the normal contralateral hips [11]. For patients who underwent bilateral THAs, there was a large variance in PT displacement, possibly due to the wide range of hip pathology that ultimately necessitated a bilateral THA [11].

This study sought to determine the significance of contralateral hip condition on changes in PT in the acute postoperative setting (1-3 months of follow-up). Institutional protocol includes obtaining 6-week x-rays and follow-up, but further follow-up/x-rays until 1 year is variable. This is the main reason why 6 weeks was chosen. However, there was subsequent analysis on all patients with further than 6-week x-rays to see if the trends persisted. The purpose of this study was to use preoperative and postoperative anteroposterior (AP) radiographs to determine if normal, arthritic, previously replaced, or bilaterally replaced hips could affect the PT in patients undergoing THA on their contralateral hip.

## Material and methods

**Figure 1:**
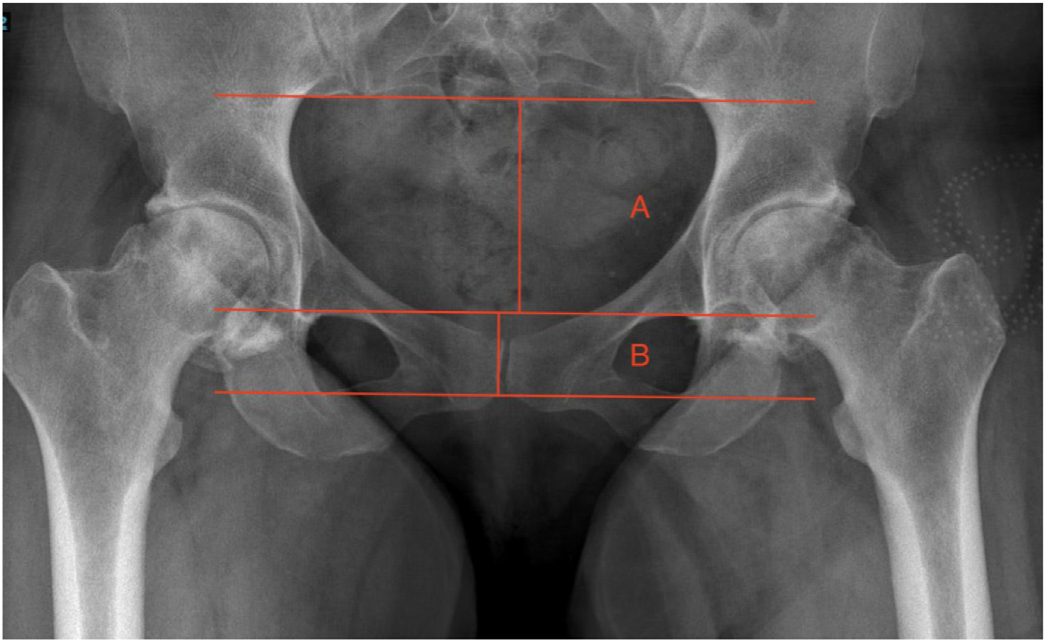
Preoperative AP plain film. Value A: Distance from the base of the sacroiliac joint to the superior margin of the obturator foramen. Value B: Height of the obturator foramen. Preoperative pelvic tilt was measured using the formula from Schwarz et al [18]: Tilt = −(ln[(B/A) × (1/0.483)]) / 0.051.

**Figure 2:**
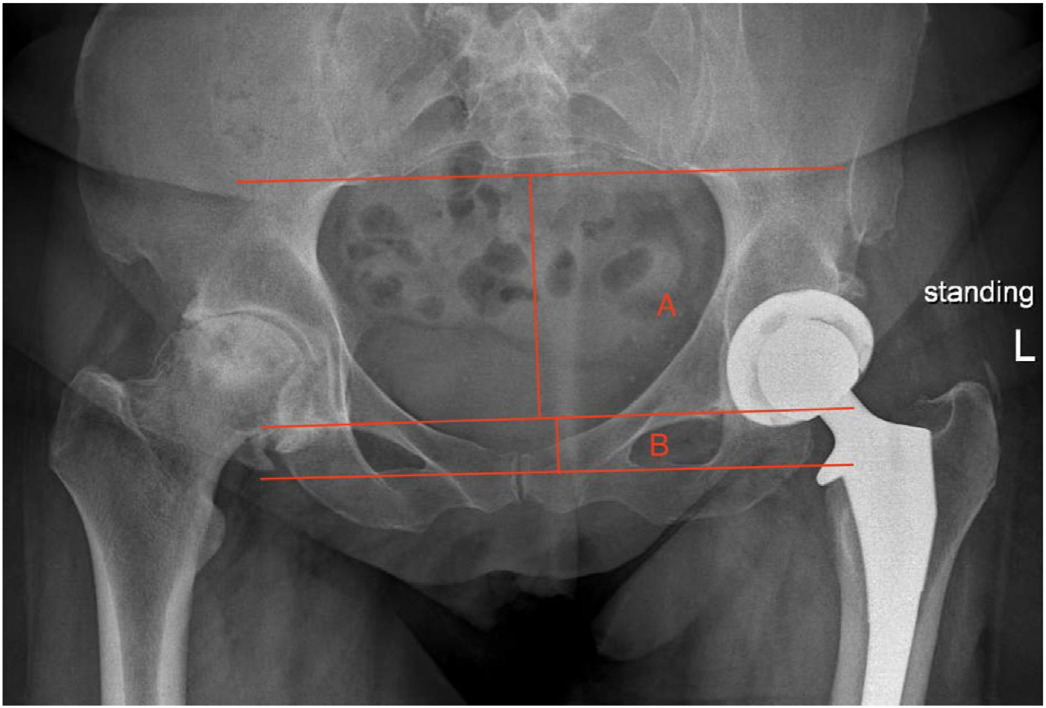
Postoperative AP plain film. Value A: Distance from the base of the sacroiliac joint to the superior margin of the obturator foramen. Value B: Height of the obturator foramen. Preoperative pelvic tilt was measured using the formula from Schwarz et al [18]: Tilt = −(ln[(B/A) × (1/0.483)]) / 0.051.

This was an institutional review board-approved retrospective study of 193 primary THAs done by 2 surgeons at a single academic tertiary referral center between September 2021 and January 2023. Forty patients were excluded for distorted anatomy, post-traumatic arthritis, insufficient x-rays, or a sacroiliac joint that could not be visualized on film. Data collected from electronic medical records included age, body mass index, and calculated PT using the formula from Schwarz et al [18], Tilt = −(ln[(B/A) × (1/0.483)]) / 0.051. PT was defined as the anterior pelvic plane and the vertical line from the symphysis. Anatomical differences between men and women’s hips were found to be insignificant in the calculation of PT; thus, the one formula was applicable to men and women. Ratios in this formula allowed for normalization of any changes in camera views of different. To compare the change in PT, standing AP preoperative imaging (Fig. 1) was compared with standing AP postoperative imaging (Fig. 2). Postoperative imaging was collected 1-3 months following surgery, and then again at least 3 months after surgery. Value A was measured as the distance from the base of the sacroiliac joint to the superior margin of the obturator foramen; value B was measured as the height of the obturator foramen. Neutral PT is determined by a tilt ratio (B/A) of 0.5, which is calculated as −0.7° in the tilt formula. More positive calculations indicate a more anterior tilt, and negative calculations indicate a more posterior tilt. Contralateral hips were identified as being normal (n = 75), arthritic (n = 39) (Tönnis grade 3/4), replaced (n = 34), or having undergone simultaneous bilateral THA (n = 5).

PT was measured in degrees with 95% confidence intervals (CIs). Differences in postoperative and preoperative PT were measured in 2 ways: mean difference and mean absolute difference. Mean difference in PT represents where the pelvis moves anteriorly vs posteriorly after THA; a negative mean difference represents increased posterior tilt vs positive mean difference represents increased anterior tilt. Mean absolute difference in PT represents the total amount of change in degrees when comparing postoperative to preoperative tilt, regardless of anterior vs posterior tilt; larger values represent greater overall change, vs smaller values represent lesser overall tilt change. Analyses for statistical significance were calculated using paired, 2-tailed *t*-tests, and one-way analysis of variance (ANOVA). Regression analysis was used to calculate correlation between contralateral hip status and mean difference and mean absolute difference in PT.

## Results

Patients with normal contralateral hips were the only group with statistically significant increased posterior tilt after THA between preoperative (0.5 95% CI [−2.0, 3.1]) and postoperative PT (−4.6 95% CI [−7.2, −2.1]) (*P* < .001) (Table 1). Patients in the arthritic and replaced contralateral hip groups also noted posterior tilting, but it was not statistically significant (*P* > .05) (Table 1). Arthritic group had a mean preoperative PT of 1.5 95% CI (−1.1, 4.0) and a postoperative PT of −0.04 95% CI (−2.4, 2.3) (Table 1). Replaced group had a mean preoperative PT of −2.7 95% CI (−5.4, 0.1) and a postoperative PT of −4.3 95% CI (−6.9, −1.7) (Table 1). Patients with bilaterally replaced hips demonstrated a nonsignificant anterior tilt after THA, with mean preoperative PT at 2.1 95% CI (−5.0, 9.2) and mean postoperative tilt at 4.3 95% CI (−2.6, 11.3) (*P* > .05) (Table 1).

**Table 1:** Demographics and mean preoperative and postoperative PT.

Contralateral side	N	Average age	Average body mass index (BMI)	Mean preoperative PT (95% CI)	Mean postoperative PT (95% CI)	*P*-value
Normal	75	61.4	32.2	0.5 (−2.0, 3.1)^a^	−4.6 (−7.2, −2.1)^a^	<.001^a^
Arthritic	39	63.1	32.2	1.5 (−1.1, 4.0)	−0.04 (−2.4, 2.3)	.13
Replaced	34	64.2	31.5	−2.7 (−5.4, 0.1)	−4.3 (−6.9, −1.7)	.08
Bilateral replacement	5	54.8	30.9	2.1 (−5.0, 9.2)	4.3 (−2.6, 11.3)	.17

*P*-values were calculated by paired 2-tailed *t*-tests between mean preoperative pelvic tilt and mean postoperative pelvic tilt.

a Denotes statistically significant difference.

Average change in PT in patients with normal contralateral hips was −5.2° 95% CI (−7.0°, −3.4°) with an absolute mean difference of 7.6° 95% CI (6.3°, 8.9°); −1.5° 95% CI (−3.5°, 0.4°) for arthritic contralateral hip with an absolute mean difference of 5.0° 95% CI (3.7°, 6.2°); −1.6° 95% CI (−3.4°, 0.1°) for replaced contralateral hip with a mean absolute difference of 4.3° 95% CI (3.2°, 5.4°); and 2.2° 95% CI (−0.4°, 4.8°) for bilateral hips with a mean absolute difference of 2.6° 95% CI (0.3°, 4.8°) (Table 2). Differences between all groups were statistically significant for mean PT change (*P* < .01) and mean absolute difference (*P* < .001) (Table 2). There were significant noncorrelations between contralateral hip status and degrees of PT change: adjusted R squared was 6.2% for mean difference (*P* < .01), and 9.8% for mean absolute difference (*P* < .001) (Table 2).

**Table 2:** Calculated degrees of difference in preoperative to postoperative PT depending on the status of the contralateral hip.

Contralateral side	Mean difference in PT (95% CI)	Mean absolute difference in PT (95% CI)
Normal	−5.2 (−7.0, −3.4)	7.6 (6.3, 8.9)
Arthritic	−1.5 (−3.5, 0.4)	5.0 (3.7, 6.2)
Replaced	−1.6 (−3.4, 0.1)	4.3 (3.2, 5.4)
Bilateral replacement	2.2 (−0.4, 4.8)	2.6 (0.3, 4.8)

Differences between all groups were statistically significant for mean difference PT (*P* < .01) and mean absolute difference PT (*P* < .001), calculated via one-way analysis of variance.

Regarding spinopelvic pathology, there were 89 (58%) total patients with noted lumbar spondylosis in preoperative AP film: 19 in the replaced contralateral hip group, 41 in the normal group, 27 in the arthritic group, and 2 in the bilaterally replaced group. All groups demonstrated a posterior PT change, except for the bilaterally replaced group, and only the normal contralateral hip group demonstrated a statistically significant change (Table 3). Mean change and mean absolute change in PT were statistically significant between all groups with noted lumbar spondylosis (Table 4).

**Table 3:** Mean preoperative and postoperative pelvic tilt for patients with lumbar spondylosis.

Contralateral side	N	Mean preoperative PT (95% CI)	Mean postoperative PT (95% CI)	*P*-value
Normal	41	5.4 (3.2, 7.6)^a^	−1.3 (−3.3, 0.7)^a^	<.001^a^
Arthritic	27	0.5 (−2.7, 3.7)	−1.1 (−3.9, 1.7)	.45
Replaced	19	−3.0 (−5.9, −0.1)	−5.0 (−8.4, −1.6)	.40
Bilateral replacement	2	−6.4 (−7.9, −4.9)	−3.6 (−12.4, −5.2)	.64

*P*-values were calculated by paired 2-tailed *t*-tests between mean preoperative pelvic tilt and mean postoperative pelvic tilt.

a Denotes statistically significant difference.

**Table 4:** Calculated degrees of difference in preoperative to postoperative PT depending on the status of the contralateral hip in patients with lumbar spondylosis.

Contralateral side	Mean difference in PT (95% CI)	Mean absolute difference in PT (95% CI)
Normal	−6.5 (−8.7, −4.3)	7.3 (5.6, 9.0)
Arthritic	−1.7 (−4.2, 0.8)	5.3 (3.7, 6.9)
Replaced	−1.9 (−4.2, 0.4)	4.1 (2.5, 5.7)
Bilateral replacement	2.8 (−4.6, 10.2)	3.8 (−1.7, 9.3)

Differences between all groups were statistically significant for mean difference PT (*P* < .01) and mean absolute difference PT (*P* = .03), calculated via one-way analysis of variance.

Of the included patients, only 7 (4.6%) had a history of lumbar fusion: 1 in the replaced contralateral hip group, 4 in the normal group, and 2 in the arthritic group. Only patients in the normal contralateral hip group noted posterior tilt after THA vs anterior changes in the arthritic and replaced groups, though none of these findings were statistically significant (Table 5).

**Table 5:** Mean preoperative and postoperative pelvic tilt for patients with a history of lumbar fusion.

Contralateral side	N	Mean preoperative PT (95% CI)	Mean postoperative PT (95% CI)	*P*-value
Normal	4	3.1	2.8	.99
Arthritic	2	−3.7	−2.1	.87
Replaced	1	−23.58	0.11	-
Bilateral replacement	0	-	-	-

*P*-values were calculated by paired 2-tailed *t*-tests between mean preoperative pelvic tilt and mean postoperative pelvic tilt.

Power analysis was performed to calculate the number of patients necessary to yield significant power for this study. With α set at 0.05 and β set at 0.2, the effect size needed to provide significant power was 74 patients in the normal contralateral hip category, 429 in the arthritic group, 395 in the replaced group, and 207 in the bilateral group. Thus, only the normal contralateral group demonstrated significant power in the study, with power calculated to be 0.81. Power was 0.13, 0.13, and 0.07 in the arthritic, replaced, and bilaterally replaced groups, respectively.

Given that 6 weeks may not have been enough time for flexion contractures to resolve, this study also further examined all patients who had a minimum of 3-month follow-up. There were 54 patients in the normal contralateral hip group, 31 in the arthritic, 24 in the replaced, and 3 in the bilaterally replaced hip group. Patients with normal, arthritic, and replaced contralateral hips demonstrated statistically significant increased posterior tilt after THA between preoperative and postoperative PT (Table 6). Patients with bilaterally replaced hips demonstrated a nonsignificant anterior tilt after THA (*P* = .20) (Table 6). While differences between all groups were not statistically significant for mean PT change (*P* = .42) and mean absolute difference in PT (*P* = .12), the same trends were noted as in 6-week x-rays. Patients with normal contralateral hips noted the largest change in posterior tilt with a mean difference in PT of −6.7 (95% CI −9.5, −3.9), followed by patients with arthritic contralateral hips noting a mean difference in PT of −6.1 (95% CI −9.1, −2.9), replaced contralateral hips noting a mean difference of −4.5 (95% CI −8/3, −0.7), and bilaterally replaced hips noting an anterior tilt of mean difference 1.8 (95% CI −0.1, 3.7) (Table 7).

**Table 6:** Demographics and mean preoperative and postoperative PT for a minimum 3 months postoperative follow-up.

Contralateral side	N	Average age	Average months of follow-up (range)	Mean preoperative PT (95% CI)	Mean postoperative PT (95% CI)	*P*-value
Normal	54	63.2	10.5 (3-27)	−0.2 (−3.2, 2.8)^a^	−6.9 (−9.6, −4.2)^a^	<.001^a^
Arthritic	31	64.0	12.6 (3-24)	1.1 (−1.8, 4.0)^a^	−5.0 (−8.3, −1.7)^a^	<.001^a^
Replaced	24	63.4	10.2 (3-18)	−2.8 (−6.0, 0.4)^a^	−7.3 (−11.4, −3.2)^a^	.03^a^
Bilateral replacement	3	53.3	8.0 (6-12)	7.8 (4.5, 11.1)	9.6 (8.2, 11.0)	.20

*P*-values were calculated by paired 2-tailed *t*-tests between mean preoperative pelvic tilt and mean postoperative pelvic tilt.

a Denotes statistically significant difference.

**Table 7:** Calculated degrees of difference in preoperative to postoperative PT depending on the status of the contralateral hip for a minimum 3 months postoperative follow-up.

Contralateral side	Mean difference in PT (95% CI)	Mean absolute difference in PT (95% CI)
Normal	−6.7 (−9.5, −3.9)	10.0 (8.1, 11.9)
Arthritic	−6.1 (−9.1, −2.9)	7.7 (5.3, 10.1)
Replaced	−4.5 (−8.3, −0.7)	7.8 (5.1, 10.5)
Bilateral replacement	1.8 (−0.1, 3.7)	1.8 (−0.1, 3.7)

Differences between all groups were NOT statistically significant for mean difference in PT (*P* = .42) and mean absolute difference in PT (*P* = .12), calculated via one-way analysis of variance.

## Discussion

Besides gait and postural differences, PT changes in the operative setting can lead to changes in proper acetabular cup positioning. Every degree of change in PT leads to a 0.7-degree change in anteversion and a 0.3-degree inclination; thus, postoperative PT changes may imply the utilization of intraoperative prophylactic compensation measures to promote accurate cup placement and prevent complications [[2], [3], [4], [5], [6], [7]].

When looking into the acute postoperative phase (1-3 months), this study noted increased postoperative posterior tilting that was only significant in patients with normal, nonarthritic, nonreplaced contralateral hips. Posterior tilting was also found in the replaced and arthritic contralateral hip groups, but was not significant. Differences in postoperative PT changes between all the study groups were significant, indicating that the status of contralateral hip could hold some influence on postoperative changes in PT.

The acute postoperative phase may not have been enough time for flexion contractures to resolve; thus, this study also examined all patients who had a minimum 3-month follow-up. Although the results were not statistically significant due to the decreased number of patients with 3-month x-rays, the same trends were still seen. Both at 6-week follow-up and at 3 months, patients in the normal contralateral hip group noted the greatest amount of posterior tilting compared to patients in the arthritic and replaced groups; though, the differences at 3 months appear to be clinically smaller. These findings were consistent with the prior notion of posterior tilting after THA [[8], [9], [10], [11],^16^], as well as the conclusions from Nishiwaki et al, who noted significantly increased posterior tilting in patients with normal contralateral hips compared to replaced and arthritic contralateral hips [11]. Further support for increased posterior tilting after THA can also be elucidated in the mean preoperative PT of the replaced group. As opposed to the other groups, patients with bilateral hips replaced in this study notably demonstrated increased anterior pelvic change in follow-ups within 3 months as well as further out, though these results may be difficult to interpret as the quantity of bilateral THAs performed were minimal. Like Nishiwaki et al, this study also noted a minimal change in preoperative to postoperative PT for bilaterally replaced patients [11].

Theories behind our results stem from the belief that the effects of hip flexor and capsular release are accentuated when the contralateral hip provides minimal resistance in preventing the uncompensated effects of the preserved hip extensor muscles, namely in the normal contralateral hip group. On the contrary, patients with a stiff contralateral hip (arthritic/replaced) provide some compensation against the strength of the hip extensors after THA [14,15]. Therefore, the first THA performed likely will not release the flexion contracture that may be present on the contralateral hip; rather, the second THA should, in theory, release the remaining contracture. This was similarly demonstrated in the results of the present study, as the replaced group continued to demonstrate a posterior tilt change after their second THA. Unfortunately, the retrospective nature of this study limits our ability to obtain lateral standing films to compare preoperative and postoperative flexion contractures of either hip. Further research will need to be conducted before solidifying the foundations of these theories [19].

Few patients in this study demonstrated a previous history of lumbar fusions, whereas more than half of them had noted lower lumbar spondylosis. Results in patients with noted lumbar spondylosis mirrored the results in the overall cohort. Patients with lumbar fusion noted less posterior tilting change when compared to the overall included patient cohort, though these results are difficult to interpret given the small sample size of the included patients with lumbar fusion. There is an understanding that patients with more significant lumbar disease have less capacity to change their PT postoperatively. Spinal pathology that leads to increased stiffness, such as scoliosis, lumbar degenerative disease, and lumbar fusion, confers increased posterior PT while in the standing position but more anterior tilt in the sitting position, likely to be caused by a lack of compensatory mechanics for proper physiologic PT change [12,[20], [21], [22], [23]]. However, for the purposes of this study, the focus was maintained on the effects of contralateral hip status.

The power of this study was largely limited by the number of included patients who received surgery by one of 2 surgeons at a single academic tertiary referral center. Limitations of this study also rest in the power of the formula used to calculate PT by Schwarz et al [18]. The formula has an approximate mismeasurement of ±4°, a validated range of −15° and +15°, and found no differences in PT between men and women, despite differences in pelvic anatomy between sexes. This study calculated PT in the acute postoperative setting; however, longer follow-up, as well as postoperative patient reported outcomes would have been beneficial in demonstrating possible late complications (eg, dislocations) and clinical significance caused by the PT changes. Although long-term effects of postoperative PT were not shown, this may have supported the findings by Katsura et al that demonstrated the progression of posterior tilting was most significant within the first year after THA, with the rate of change of PT slowly stabilizing after the 1-year mark [10], while another study by Blondel et al found no significant variation in PT after 3-year follow-up [13]. It is also important to note that in this study, changes to PT after THA were not predicted. Rather, intraoperative PT was matched as closely as preoperative imaging, with cups placed at approximately 40-45 degrees and 20 degrees of anteversion. Nonetheless, these results may affect how cups are placed in the future by these surgeons. Lastly, spinopelvic influence on PT changes postoperatively was not clearly delineated as the subject of this study focused on effects from the possible influence from the contralateral hip, and thus limited data was retrieved on spinal pathology (eg, lack of full-length spine plain films).

There are a number of algorithms, both in the published literature and commercial products that help take into account PT to optimize cup position, but these are using the current state of the pelvis as the model and do not take into account the possible postoperative changes in PT [[24], [25], [26], [27], [28], [29]]. Though on average the postoperative PT change was small, there are some patients with large enough tilt changes to have clinically meaningful effects on acetabular cup position. Further studies may help delineate which patients are at risk for larger pre-to-post changes in PT. Based on present data, there is typically more posterior tilt after THA; hence, surgeons may air on the side of slightly less anteversion and inclination on cupplacement.

## Conclusions

Contralateral hip status has the potential to influence PT changes in the postoperative setting. However, many studies will need to continue investigating the possible predictive degrees of change before prophylactic intraoperative PT measures are taken into effect to mitigate the posterior tilting after THA. Though spinopelvic relationships also play an important factor in the postoperative effects on PT, more attention was placed on the postoperative effects of contralateral hip status. It will be useful for future studies to investigate if the extent of arthritis or lumbar fusion affects postoperative PT change. It will also be increasingly useful to determine the clinical significance of these postoperative changes, including postoperative complications, postural, or gait changes.

## Conflicts of interest

D. Schmitt is a paid consultant for HipInsight. J. Pierrepont is a paid consultant and has stock options in Corin Group. N. Brown receives royalties from Corin and Link; is a paid consultant for Depuy, Corin, Link, and Smith and Nephew; and is a board/committee member of AAOS. All other authors declare no potential conflicts of interest.

For full disclosure statements refer to https://doi.org/10.1016/j.artd.2024.101460.

## CRediT authorship contribution statement

**William Oetojo:** Writing – review & editing, Writing – original draft, Methodology, Investigation, Formal analysis, Data curation. **Patrick Lawler:** Writing – review & editing, Methodology, Investigation, Data curation. **James Padley:** Writing – review & editing, Methodology, Data curation. **Jim Pierrepont:** Writing – review & editing, Validation, Supervision, Conceptualization. **Daniel Schmitt:** Writing – review & editing, Validation, Supervision, Project administration, Conceptualization. **Nicholas Brown:** Writing – review & editing, Validation, Supervision, Project administration, Investigation, Conceptualization.

## Appendix A Supplementary Data

Conflict of Interest Statement for Padley

Conflict of Interest Statement for Pierrepont

Conflict of Interest Statement for Lawler

Conflict of Interest Statement for Oetojo

Conflict of Interest Statement for Brown

Conflict of Interest Statement for Schmitt
